# Bacteriophages: a double-edged sword in the gastrointestinal tract

**DOI:** 10.3389/frmbi.2024.1450523

**Published:** 2024-09-23

**Authors:** Yuqi Wei, Chunli Zhou

**Affiliations:** Department of Gastroenterology, The Affiliated Suzhou Hospital of Nanjing Medical University, Suzhou Municipal Hospital, Gusu School, Nanjing Medical University, Suzhou, Jiangsu, China

**Keywords:** bacteriophages, gut microbiome, phage–bacteria interaction, phage-related diseases, phage therapy

## Abstract

The symbiotic relationship between the gut microbiome and the human body is a concept that has grown in popularity in recent years. Bacteriophages (phages) are components of the gut microbiota and their imbalance plays a role in the pathogenesis of numerous intestinal disorders. Meanwhile, as a new antimicrobial agent, phage therapy (PT) offers unique advantages when compared with antibiotics and brings a new dawn for treatment of multidrug-resistant bacteria in intestinal and extraintestinal disorders. In this review, we provide a brief introduction to the characterization of phages, particularly focusing on newly discovered phages. Additionally, we outline the involvement of gut phages in disease pathogenesis and discuss the status and challenges of utilizing phages as therapeutic targets for treatment of enteric infection.

## 1 Introduction

In the natural world, viruses are the most abundant biological entities, outnumbering their cellular hosts by a significant margin (Edwards and Rohwer, 2005). As the most prevalent type of virus, phages are ubiquitous in the gut of humans and display a source of vast genetic diversity (Dutilh, 2014). According to data from the Global Virome Database, phages comprise 97.7% of the human gut virome, which is dominated by members of *Caudovirales* and *Microviridae* (Hoyles et al., 2014; Gregory et al., 2020; Sausset et al., 2020). The first viral metagenomic analyses of feces samples revealed that most viral genomic sequences (59%) did not match any known viruses (Breitbart et al., 2003), meaning that our current level of knowledge of intestinal phages is limited.

Over recent decades, most studies have closely linked bacterial communities with gut diseases, neglecting the influence of phages. Indeed, in the analysis of meaningful microbiota changes in many diseases, significant changes in phage composition are frequently observed, suggesting a potentially crucial role for phages in disease pathogenesis. Besides, with the rampant prevalence of multidrug-resistant microorganisms worldwide, there is an urgent demand for the development of antimicrobials with precise target specificity. In this context, phages have received considerable attention as a viable alternative or aid for infectious diseases in place of antibiotics.

This review summarizes the evidence of the roles of gut phages in physiology and pathology. Besides, phages are proposed for disease diagnosis, gene delivery, and food safety testing, serving as an alternative to antibiotics in sterilization and more (Rasmussen et al., 2020a; Nath et al., 2022). A greater understanding of phage biology will contribute to utilization of phages as a novel frontier for disease diagnostics and treatment.

## 2 Phages in the healthy human gut

Of the phages that infect bacterial cells, most are categorized as virulent or temperate based on their strategy for host interaction (Hobbs and Abedon, 2016). The initial gut virome in infants is dominated by active temperate phages derived from the induction of prophages in early colonizing gut bacteria. As time progresses, the overall composition of virus in the infants undergoes significant changes (Gabisoniya et al., 2016). By the time children reach the age of 3 years, the viral population of healthy adults has roughly taken shape. Phages in the distal gut attain greater abundance during adulthood, primarily comprising a population of stable and highly specialized temperate phages (Shkoporov and Hill, 2019; Shkoporov et al., 2019). Manrique et al. put forward the concept of “core bacteriophages”, which consists of phages present in more than half of all healthy individuals. They deduced that core bacteriophages play a role in maintaining human health because of their significant reductions in patients with ulcerative colitis (UC) and Crohn’s disease (CD) (Manrique et al., 2016). Intestinal phages also vary significantly in terms of geography and ethnicity, which may be related to dietary differences (Zuo et al., 2020).

Metagenomic sequencing has resulted in a fundamentally refined perception into phages, exemplified by the discovery of crAssphage. In 2014, reassembly of most of the known viral metagenomes led Dutilh et al. to the discovery of a novel bacteriophage family known as “crAssphage” (Dutilh et al., 2014). Most crAssphage-encoded proteins do not match any known database sequences in the database (PhAnToMe, http://www.phantome.org/). Therefore, crAssphage does not fit the usual classification. Three years later, Yutin et al. used sensitive protein sequence analysis methods to re-analyze the genomic and metagenomic databases and discovered a previously unidentified, sizable, and varied family of bacteriophages known as crAss-like phage that bears striking similarities to crAssphage (Yutin et al., 2018).

Electron microscopic examination of CsCl purified concentrate from a CrAss-like Phage-Rich Fecal Filtrate revealed that viral particles are largely dominated by *podoviruses* (53%), and the relative abundance of *Siphoviridae* is low (15%) (Guerin et al., 2018). Prototypical crAssphage (p-crAssphage) is made up of a double-stranded DNA genome (97 Kb) and a short-tailed virion that resembles those of *Podoviridae*. Analysis of the metagenomes for this phage family revealed the presence of genes associated with lysogenic and lytic pathways. Through sequence alignment of the TerL protein, crAss-like phages were classified into five subfamilies (alpha-gamma, beta, delta, epsilon, and zeta) and ten candidate genera, of which the p-crAssphage belongs to subfamily *Alphacrassvirinae *(Guerin et al., 2018; Yutin et al., 2021) (https://ictv.global/taxonomy). It is widely accepted that the phylum *Bacteroidetes* is probably its host. Zheng et al. were the first to use a novel high-throughput method (Microbe-seq) to show that there is a notable *in vivo* host–phage association between crAssphage and *Bacteroides vulgatus* (Zheng et al., 2022). By studying gut phage lineages, *Bacteroides*-associated carbohydrate-binding often N-terminal (BACON) domains in crAssphage were found to assist crAss-like phages in binding to the *Bacteroides* cell surface, providing further supporting evidence of this viewpoint (Jonge et al., 2019). As one of the oldest phages to have co-evolved with humans, crAssphage has been found in roughly half of human gut viromes and accounted for 86.7% of the sequence reads from virus genomes, dominating the human gut virome (Dutilh et al., 2014). Furthermore, allogeneic crAssphage was found to persist for 1 year in recipients following successful fecal microbiota transplantation (FMT) (Draper et al., 2018). Thus, crAssphage may also belong to the core human gut phageome. New metagenomic studies demonstrated that the genomes of this group are widespread across the globe, including the animal gut and aqueous environments (Yutin et al., 2018; Li et al., 2021; Sabar et al., 2022). Many studies assessing the influence of genetic and environmental factors on crAssphage abundance in the gut showed that it did not show a significant association with many diseases (such as diarrhea and metabolic syndrome) and gender, but was correlated rather with dietary habits and industrialized lifestyle (Liang et al., 2016; Edwards et al., 2019; Honap et al., 2020; de Jonge et al., 2022). Besides, in a gut metagenomic analysis of 1,950 individuals from the Netherlands, the crAss-like phage was observed with a degree of stability over 4 years but showed depletion in patients with inflammatory bowel disease (IBD) (Gulyaeva et al., 2022). Interestingly, the abundance of crAssphage family members has been reported to be markedly higher in patients diagnosed with UC compared to healthy controls (Yang et al., 2020). The huge variation in the richness of this phage in the gut and differences in the reference databases used may cause the differences between these results. In exploring the possible applications of crAssphage, because of its highly group-specific expression and wide distribution, crAssphage has the potential for development as a microbial source tracking (MST) marker of human fecal contamination (Sabar et al., 2022).

Although crass-like phage genomes are abundant in the human gut, there are only a small number of species successfully isolated because of the scarcity of pure isolates. The first member of the crAss-like group, named ΦcrAss001, was isolated by Shkoporov et al (Shkoporov et al., 2018). Despite being a virulent phage, it has been demonstrated that ΦcrAss001 can coexist with its host cells for a long term due to the following two mechanisms: (i) rapid phase variation of capsular polysaccharide leading to phage resistance in host cells occurs stochastically; (ii) infected cells not lysis promptly due to pseudolysogeny or the formation of a carrier state (Shkoporov et al., 2021). In addition, Hryckowian et al. successfully isolated two novel species infecting *Bacteroides thetaiotaomicron* VPI- 5482, named *Wulfhauvirus bangladeshii* DAC15 and DAC17 (Hryckowian et al., 2020). In 2020, researchers discovered ΦcrAss002, which belongs to the proposed *Alphacrassvirinae* subfamily and infects *Bacteroides Xylanisolvens* (Guerin et al., 2021). In co-culture media, this crAss-like phage may coexist with its hosts for extended periods of time, reaching large titers similar to ΦcrAss001. Recently, three novel crAssphage species infecting *Bacteroides cellulosilyticus* WH2 and 25 new crAss-like phages (termed crAssBcn) have been isolated from wastewater (Papudeshi et al., 2023; Ramos-Barbero et al., 2023). In comparing the genomes of the crAssBcn phages, researchers found that DNA polymerase as the traditional target gene for phage detection was highly variable, thus they suggested searching for other more conserved genes like terminase, or RNA polymerase (Guerin et al., 2018; Ramos-Barbero et al., 2023).

## 3 Phage-mediated immune responses

It is estimated that nearly half of the bacterial genomes in the human gut contain at least one phage genome (Touchon et al., 2016). These integrated phage DNA sequences, called prophages, originate from temperate phages before they enter the lytic cycle (Howard-Varona et al., 2017). Prophages can also exist within a host bacteria extrachromosomally. *Escherichia coli* phage P1, which has been widely used in biological studies, exists as a plasmid in a circular form (Åobocka et al., 2004). The choice of lysis versus lysogeny by temperate phages weighs the cost of a delayed lysis cycle against the impact of environmental stress (Goldhill and Turner, 2014). Under situations of low bacterial density, low nutritional concentration, and low temperature, lysogens are more prevalent (Shan et al., 2014). Changes in the intestinal environment under some disease states are thought to be another major factor in the activation of prophages (Oh et al., 2019). Aberrant inflammation induces the activation of the RecA protein and triggers the bacterial SOS response system, which manages a coordinated response to DNA damage, thereby generating free bacteriophages (Diard et al., 2017). In addition, prophages can be induced spontaneously or through alternative pathways (Bertani, 1951). For example, in a co-culture system, production of Lambda phage in *recA-*deficient *E. coli* was associated with acyl-homoserine lactones produced by *Pseudomonas aeruginosa* (Ghosh et al., 2009). Prophages exert a multifaceted influence on the survival strategies of their host organisms, which ultimately provide advantageous adaptations for the bacterial hosts. Genes carried by virions (such as those involved in antibiotic resistance, metabolism, and bacterial virulence) are horizontally transferred to other bacteria, which may then adopt entirely new phenotypes (Johnson et al., 2017; Touchon et al., 2017). Haaber et al. demonstrated that phages spontaneously released by *S. aureus* constantly propagate, with these viral transduction particles capturing DNA fragments from competing cells killed by the phages. This process, known as “autotransduction”, enables lysogenic bacteria to acquire resistance genes (Haaber et al., 2016). Additionally, prophages, along with the products encoded by their genes, facilitate bacterial host diversification and confer host-selective advantages. Studies on colicin Ib have shown that the presence of temperate phages and their lysis genes confer distinct advantages for bacterial hosts in competition with Collb-susceptible competitors (Nedialkova et al., 2016).

Direct or indirect interactions between phages and gut cells will antiviral immune responses, inducing anti-inflammatory and proinflammatory effects. Phages can bypass intact epithelial layers, thereby establishing direct contact with immune cells located beneath the intestinal lining (Nguyen et al., 2017). Intriguingly, phage richness in mucosal surfaces from invertebrates and vertebrates was found to be higher than that in the adjacent environment. *In vitro* experiments demonstrated that weak binding interactions mediated the enrichment of phages between mucin glycoproteins and immunoglobulin-like domains exposed on the phage capsid (Barr et al., 2013). Barr et al. described the mechanism of this phenomenon and developed the BAM model, which posits that bacteriophages adhering to mucus can protect metazoans from external bacterial infections. This model was later expanded to the “tripartite symbiosis” model, which shows the beneficial interactions among intestinal epithelial cells, bacteria, and phages, maintaining gut and overall homeostasis. Bacteriophages act on epithelial cells to participate in mucus layer production and coordinate the secretion of metabolic products. These metabolic products serve as a nutrient source for intestinal symbiotic bacteria, ensuring that these bacteria become the dominant flora and potential hosts for bacteriophages. The interactions among these three components establish a positive feedback loop, maintaining intestinal health (Barr, 2019). Chin et al. used a gut-on-a-chip system to simulate the intestinal mucosa and found that selective pressures, such as mucus turnover and glycosylation, promote beneficial phage mutations and phage-bacteria coevolution. Phages achieve their competitive advantage by rapidly mutating the Ig-like binding domains of their proteins, altering the affinity of the capsid protein Hoc for mucin glycans (Chin et al., 2022). These tripartite evolutionary interactions maintain a long-term, stable, and nuanced equilibrium in the gut.

Changes in phage-mediated lysis of bacteria lead to modulated concentrations of many bacterially-derived metabolites, including bacterial DNA and lipopolysaccharide (LPS). The release of pathogen-associated molecular patterns (PAMPs) participates in a feedback loop between phage induction and intestinal inflammation, known as the “positive inflammatory feedback loop” (Jansen and Matthijnssens, 2023). Besides, phages directly activate the immune system of mammals through recognition by Toll-like receptor (TLR)9, inducing interferon (IFN)-γ responses and exacerbating colitis. This is corroborated by the observation of marginal increases in the percentage of CD4+ and CD8+ T cells in the mesenteric lymph nodes (MLNs) in rodents treated with phage (Gogokhia et al., 2019). Additionally, Liu et al. found that the symbiotic viruses via noncanonical Retinoic Acid-inducible Gene-I (RIG-I) signaling can restore homeostasis of lamina propria lymphocytes and reverse the susceptibility to dextran sulfate sodium-induced colitis in mice (Liu et al., 2019).

It is estimated that 3.1 × 10^10^ phages are absorbed daily from the intestine in humans, reaching multiple parts of the body – e.g., cerebrospinal fluid (CSF), lung, pericardium, and urine – through the lymphatic and circulatory systems, and finally being eliminated by the mononuclear phagocytic system (MPS) in the liver and spleen (Nguyen et al., 2017). When the host organism encounters the foreign phage particle, phage triggers immunological reactions, including phagocytosis, a respiratory burst of phagocytic cells, anti-phage humoral responses, and T-cell proliferation (Van Belleghem et al., 2018). When peripheral blood mononuclear cells (PBMC) are stimulated by phages, both anti-inflammatory response by up-regulating anti-inflammatory gene expression (*IL1RN*, *IL10*, and *SOCS3*) and down-regulating pro-inflammatory gene expression (*CXCL1*, and *CXCL5*) and inflammatory response by up-regulating inflammatory gene expression (IL-1 α, *IL-1 β*, and *TNF- α*) were present. Among them, anti-inflammatory effects have a predominant role, which may decrease phage clearance or inactivation from human bodies (Van Belleghem et al., 2017). Besides, phages have been proven to reduce the release of reactive oxygen species (ROS), a substance that indicates inflammation. T4 phage-mediated inhibition of ROS formation is achieved by diminishing the LPS-mediated stimulation of peripheral blood polymorphonuclear leukocytes (McManus and Heneka, 2017).

## 4 Phages and their applications in disease

### 4.1 Inflammatory bowel disease

IBD is a non-specific, chronic, inflammatory intestinal disease that includes two conditions: CD and UC (Kaplan and Ng, 2017). Data from numerous studies have shown dysbiosis of phage community composition in patients with IBD, characterized by a higher richness and abundance of *Caudovirales* (**Table 1**) (Wagner et al., 2013; Norman et al., 2015). Additionally, a study of CD patients reported that the number of virus-like particles was significantly increased in the intestinal lumen and decreased in areas of ulceration on mucous membranes, indicating that the loss of mucosal phages acting as sentinels may underlie chronic intestinal inflammation in CD (Cao et al., 2024). Contrary to previous studies, Clooney et al., who utilized whole-virome analysis of two IBD virome datasets, concluded that the virome’s overall richness did not differ significantly between health and IBD. They observed an increase in temperate phage sequences, which replaced the healthy core of virulent phages in IBD patients (Clooney et al., 2019).

**Table 1:** Summary of overall changes to the gut phageome/virus and alterations in specific phages/virus in different disease states.

Disease	Overall changes	Alterations in specific phages	Reference
CD	increase in phage richness	increase in *Caudovirales*	(Wagner et al., 2013)
CD	increase in phage richness and diversity	–	(Pérez-Brocal et al., 2015)
UC	decreased mucosal virome diversity and richness	a significantly higher abundance but lower diversity, richness, and evenness of *Caudovirales*; enriched in *Escherichia* phage and *Enterobacteria* phage	(Zuo et al., 2019)
IBD	increase in phage richness and diversity	increase in *Caudovirales*	(Norman et al., 2015)
IBD	no significant differences in phage richness and diversity	significantly lower in the number of *Microviridae* phage	(Fernandes et al., 2019)
CDI	–	a significantly higher abundance but lower diversity, richness, and evenness of *Caudovirales*; lower abundance and diversity of *Microviridae*;	(Zuo et al., 2018)
T1DM	no significant differences in global viral composition	six virus taxa belonging to the family *Ruminococcaceae* and family *Lachnospiraceae* are differentially abundant	(Hu et al., 2023)
T1DM	Lower phage Shannon diversity and richness	significant enrichment of *Circoviridae*-related sequences	(Zhao et al., 2017)
T1DM	–	differential abundance enriched in 25 phage types	(Clos-Garcia et al., 2022)
T2DM	no changes in abundance of the phage families	significantly increase in *Enterobacteriaceae*-specific phages	(Chen et al., 2020)
T2DM	A significant increase in the number of gut phages but no significant difference in phage abundance	significantly increase in the relative numbers of the *Myoviridae*, *Podoviridae*, *Siphoviridae*, and unclassified-*Caudovirales* families	(Ma et al., 2018)
Obesity and T2DM	decrease in viral richness and diversity, and more pronounced in T2DM	enriched in 11 viruses, and relative abundance of *Escherichia* phage, *Geobacillus* phage, and Lactobacillus phage are the largest	(Yang et al., 2021)
T2DM	A significant decrease in viral richness and diversity, and more pronounced in T2D-Ne	63 phage species significantly changed in T2D; 2 viral orders, 9 families and 8 genera are significantly decreased, while 9 genera are increased in T2D-Ne	(Fan et al., 2023)
irritable bowel syndrome (IBS)	no changes in alpha and beta diversity of phage	*Microviridae*, *Myoviridae*, and *Podoviridae* species are elevated in IBS-Diarrhea, and other *Microviridae* and *Myoviridae* species are elevated in IBS-Constipation	(Mihindukulasuriya et al., 2021)
CRC	A significant increase in phage richness and diversity	enriched in members of *Inovirus* and *Tunalikevirus*	(Nakatsu et al., 2018)
CRC	Higher virome alpha diversity and evenness	enriched in 11 viral species from *Podoviridae*, *Siphoviridae*, and *Myoviridae* specie, while depleted in *Herelleviridae*	(Zuo et al., 2022)
CRC	No differences in viral richness or Shannon or Simpson indexes	enriched in *Escherichia* viruses and *Salmonella* viruses, while depleted in *Enterobacteria* phages and Uncultured crAssphage	(Gao et al., 2021)
alcoholic hepatitis	A significant increase in viral richness and diversity	*Escherichia*-, *Enterobacteria*-, and *Enterococcus* phages are overrepresented	(Jiang et al., 2020)
NAFLD	the average relative abundance and viral diversity of phages decreased significantly with the progression of hepatic fibrosis	significantly less in *Lactococcus* phages and significant increase in *Streptococcus* TP-J34 phages in patients with advanced stage of NAFLD and severe fibroses	(Lang et al., 2020)
MetS	lower viral richness and diversity of gut viromes	enriched in phages infecting *Streptococcaceae* and *Bacteroidaceae* and depleted in those infecting *Bifidobacteriaceae*	(de Jonge et al., 2022)
atherosclerotic cardiovascular disease	a significant increase in viral richness at the family level and a visible alteration in overall virome structure	105 viral operational taxonomic units increased in abundance	(Li et al., 2024)
amyloid-positive Alzheimer’s disease	a lower richness	Significant decreases of *Siphoviridae* family and *Lactococcus* phages	(Ghorbani et al., 2023)

To assess the impact of phage community alterations during IBD, the researchers simulated the expansion of free phages in a colitis mouse model. Significant weight loss and more pronounced inflammation were observed in the animals with the amplified phages compared to the control, suggesting that localized increases in phage abundance in patients with IBD contribute to disease severity (Gogokhia et al., 2019). Sinha et al. gave human microbe-associated (HMA) mice fecal virus-like particles (VLPs) from UC patients and healthy individuals. Animals were subsequently given 2% DSS to induce colitis. Mice administered UC VLPs developed more severe disease, characterized by weight loss, a significant reduction in colon length, and an increase in pro-inflammatory factors, compared to mice administered healthy VLPs (Sinha et al., 2022). Another experiment confirmed that ileal mucosal viral particles from CD patients also lead to increased intestinal inflammation in a mouse model of IBD. These changes may be caused by the restructuring of the gut virome-bacteriome ecology prior to intestinal inflammation and the enhancement of microbial defense pathways in the intestine cells (Cao et al., 2024). Besides, it is widely acknowledged that *Faecalibacterium prausnitzii*, which produces butyrate and microbial anti-inflammatory protein to exert strong anti-inflammatory activities, has low abundance in the gut microbiota of IBD patients (QuÃvrain et al., 2016; Rossi et al., 2016; Cornuault et al., 2018). Cornuault et al. showed that prophages are pervasive in *F. prausnitzii* genomes and that certain phages are more commonly found in IBD patients than healthy controls, with probable positive effects on inflammatory activity (Tetz and Tetz, 2016).

In the treatment of IBD, FMT is considered one of the alternatives to standard therapy. The researchers found that UC patients with a lower relative abundance of Caudovirales phages exhibited a more pronounced effect after FMT transplantation. A specific subset of phages of donor origin persisted in IBD patients who did not respond to FMT, indicating that certain phages may impede the effectiveness of FMT by directly affecting the composition of beneficial bacteria in the microbiota (Gogokhia et al., 2019).

### 4.2 *Clostridioides difficile* infection

*C.difficile* is an opportunistic pathogen and is the leading cause of antibiotic-associated diarrhea in developed countries (Freeman et al., 2010). Genomic analysis illustrates that phage genes are ubiquitous among *C. difficile* genomes and mediate the transcriptional regulation of various genes related to pathogenicity in bacteria (Fortier, 2018; RamÃ­rez-Vargas et al., 2018). Such phages mostly belong to the order *Caudovirales*, family *Myoviridae* or *Siphoviridae*, and display a temperate lifestyle (Fortier, 2018; Heuler et al., 2021). However, only a few *C.difficile* infection (CDI) studies have delved into the dynamics of CDI phages and their correlation with CDI (**Table 1**).

Clinical data indicate that the rate of incidence and recurrence of CDI is high, and the antibiotic therapeutic effect is not promising. As an established treatment for recurrent CDI, FMT has good outcomes, apparent safety, and cost-effectiveness, which was written into the latest clinical guidelines of CDI. By comparing the viral composition of CDI patients before and after FMT, researchers found that the recipient’s phages were replaced by donor phages post-FMT, and that patients who responded to FMT had a higher *Caudovirales* richness of donor phages (Zuo et al., 2018). Similar connections between the patient’s gut virome and the donor’s gut virome several months following FMT treatment for CDI were described by Broecker et al (Broecker et al., 2017). With the deepening of research, researchers gradually realized that bacteria were not the only effective transferred component. The non-bacterial gut inhabitants, including phage, are beneficial for the reconstruction of a healthy ecological environment after FMT treatment. Five patients with symptomatic chronic-relapsing CDI who received fecal filtrate transfer (FFT) treatment experienced symptom relief for at least 6 months and produced phages similar to the donor fecal microbiota community within 6 weeks (Ott et al., 2017). In subsequent research, fecal virome transplantation (FVT, sterile-filtered feces) has been shown to promote the colonization of probiotics and the recovery of antibiotic-damaged bacterial communities (Draper et al., 2020; Rasmussen et al., 2023).

### 4.3 Colorectal cancer

It is estimated that dysbiosis, an important contributor to the progression of colorectal cancer (CRC), is present in approximately one-third of CRC patients (Marongiu and Allgayer, 2022). In analyses of stool samples from CRC patients, expansion of the alpha diversity and evenness of the virome have been observed, which are closely associated with clinical stages of patients with CRC (Nakatsu et al., 2018). Through random forest modeling, Hannigan et al. found that temperate phages represented a major component of the cancer-associated virome, and hypothesized that the influential phages were central members within the bacterium-virus community network. Accordingly, they put forth a model conceptual model aimed at elucidating the mechanisms underlying the role of phages in CRC progression. Phages disrupt the balance of non-inflammatory gut intestinal microbiota, establishing a biofilm made of cellular lysates. This creates a favorable environment for colonization and reproduction of colonized oncogenic bacteria (Hannigan et al., 2018). Although the investigation is a proof-of-concept, it highlights the irreplaceable role of complex interactions between bacteria and phages in CRC pathogenesis. By setting up a longitudinal study in CRC mouse model, researchers found that different genera of phages may play opposite roles in carcinogenesis. For example, the growth of tumors showed positively correlated with *Brunovirus* and *Hpunavirus*, but negatively correlated with *Lubbockvirus* (Li et al., 2022). Metagenomics analysis showed that 12 weeks and 24 weeks after *H. pylori* infection, compared with the wild-type mouse model, there is a significant increase in tumor number and specific vOTUs (mainly temperate phages) in tumor-prone Apc+/1638N mouse model. Studies have further revealed the association between this phenomenon and bacterial infections. The induction of prophages triggered by *H. pylori* infection contributes to CRC development in mice through several mechanisms: 1) increase in bacteria associated with CRC; 2) loss of symbiotic bacteria protection; 3) pattern-triggered immunity due to cell lysis; 4). directly trigger the immune response. In addition, there are several other potential ways that have not been explored, including a general induction pattern in intestinal bacteria and virulence gene transmission (Luo et al., 2023).

### 4.4 Liver diseases

Recent studies have highlighted the imbalance in the interspecies interactions of the gut microbiota influences liver disease through the gut–microbiome–liver axis (Chassaing et al., 2014; Suk and Koh, 2022). According to a study conducted by Jiang et al., alcohol-associated liver disease was associated with increased viral diversity in fecal samples, especially those collected from patients with alcoholic hepatitis compared to those from healthy controls. Besides, they found that specific viral taxa (*Staphylococcus* phages) were involved in disease severity and fatality (Jiang et al., 2020). In contrast, patients with nonalcoholic fatty liver disease and fibrosis were observed to have significantly reduced enterovirus diversity (Lang et al., 2020). Alcohol use was also shown to alter enterovirus yield, with lower relative abundances of phages from *Propionibacterium*, *Lactobacillus*, and *Leuconostoc* (Hsu et al., 2022). These alterations were rapidly reversed in patients who stopped drinking. Through bioinformatics strategies, the researchers assembled core phages for different etiologies of cirrhosis, with crAssphage being the most prevalent strain in all cohorts. They also analyzed differences in the presence of intestinal phages in cirrhosis cohorts from different regions, suggesting that intestinal phages could be strongly affected by geographic, ethnic, or lifestyle differences (Naseri et al., 2022). Additionally, a separate study noted a decline in crAssphage abundance with the progression of liver cirrhosis, correlating closely with patient prognosis (Bajaj et al., 2021). This suggests that complex interactions between bacteria and phages may be intertwined with treatment responses and disease advancement (Bajaj et al., 2021). Subsequent investigations are needed to fully disentangle the correlation between the downward trend in phage–bacteria interaction and liver disease.

### 4.5 Diabetes

Presently, it is well acknowledged that enteroviral infection serves as a key environmental factor leading to islet autoimmunity and subsequent diabetes in predisposed individuals. Enteroviruses reach the pancreas by hijacking cellular vesicular trafficking mechanisms, and virus responses trigger circulating “cytokine storms”, resulting in intra-islet inflammation that may ultimately result in β-cell damage (Faulkner et al., 2021; Krogvold et al., 2022). In addition, phages play a unique role in pancreatitis. In the early stages of disease progression, the die-off of high amyloid-producing *E. coli* caused by the induction of prophages led to the release of amyloid aggregates into the biofilm. Amyloid aggregates are thought to be implicated in the activation of the immunological pathway in children at risk for T1DM (Tetz et al., 2019). By inhibiting the mangiferic acid pathway and reducing the production of *Akkermansia muciniphila*, *Lactobacillus johnsonii*, as well as butyrate, phages indirectly trigger bystander CD8+ T cell activation, leading to autoimmunity (Anderson, 2023). Furthermore, inhibition of the mangiferylate pathway may be linked to pancreatic β-cell apoptosis (Hansen et al., 2012; Mesnage and Antoniou, 2020). In a prospective longitudinal study involving infants at risk for Type 1 Diabetes mellitus (T1DM), researchers recruited 11 infants possessing HLA risk genotypes. Among them, five infants developed T1DM (case group), while six did not (control group). Through comparative analysis of the enterovirus groups, researchers observed a significantly lower Shannon diversity of phages in the case group. Importantly, these alterations were detected prior to the onset of autoimmunity associated with T1DM. This finding suggests that the enteroviruses may exert a more significant influence on T1DM development compared to bacteria, as changes in bacterial composition typically occur after the emergence of islet-specific autoantibodies (Zhao et al., 2017; Park and Zhao, 2018). In a cross-sectional study, the strength of correlations between bacterial and virome communities in the adult-onset T1DM groups was significantly weaker compared to healthy controls. The majority of the reduced positive correlations involved beneficial bacteria and bacteriophages, specifically from the *Podoviridae* or *Siphoviridae (* Hu et al., 2023).

Type 2 diabetes mellitus (T2DM) is part of the metabolic syndrome, prominently characterized by insulin resistance. Among the existing research, the results of overall phage alterations in T2DM are inconsistent or contrary (**Table 1**). Ma et al. used metagenomic sequencing to catalog gut phages from fecal samples collected from T2DM patients. They found that the composition of the gut phageome was significantly altered in T2DM, identifying seven T2DM-specific core phage taxa in more than two-thirds (n > 96) of the samples (Ma et al., 2018). Furthermore, they observed significant symbiotic/exclusionary interactions between phages and other members of the gut microbiota (Ma et al., 2018). Interestingly, weight gain and glycemic abnormalities induced by a high-fat diet in mice were significantly mitigated following fecal viroid transplantation (FVT). This phenomenon is hypothesized to stem from phage-mediated alterations in the composition of the host flora in the mouse gut (Rasmussen et al., 2020b). The complex phage–bacteria interactions present an innovative pathway for future investigations into the underlying mechanisms of T2DM. Recent studies have shown that intestinal Gram-negative bacterial lysis by phages leads to the elevation of circulating LPS content in patients, inducing chronic subclinical systemic inflammation and insulin resistance (Mehta et al., 2010; Chen et al., 2020). The elevated levels of serum LPS, IL-6, and TNF-a observed in patients with T2DM support this speculation (Chen et al., 2020).

### 4.6 Causal inferences between phages and disease

**Figure 1:**
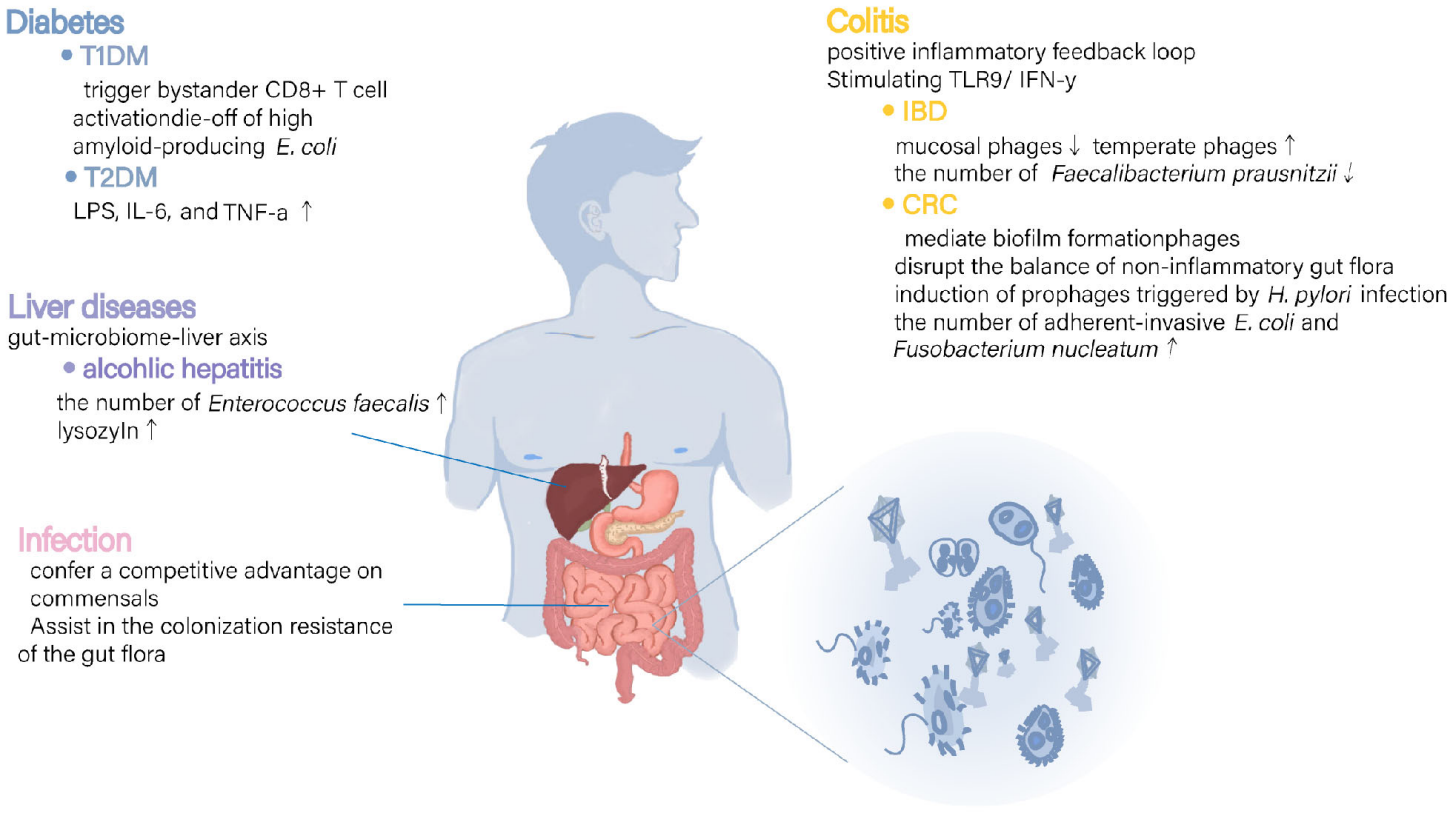
Mechanisms of possible involvement of intestinal phages in various diseases. In various diseases a, disorganization of intestinal phages has been linked to changes in immune and inflammatory response regulators as well as alterations in bacterial populations and their metabolites. These findings suggest that phages may play a role in disease processes by directly modulating immune responses and undirectly regulating bacterial community metabolism.

Growing evidence indicates that gut-associated phages can significantly influence gastrointestinal pathophysiology by regulating the intestinal immune response and network interactions among intestinal bacterial communities (**Figure 1**). Studies investigating the gut microbiota and disease commonly report fluctuations in gut phage abundance. However, because of the intricate polymicrobial ecology in the human gut, research has not arrived at a consensus on the role of phage in diseases: whether it represents a causal factor, a coincidental risk factor, a compensatory response, or merely an epiphenomenon in response to the gut disease (Guzzo et al., 2022). Future longitudinal investigations are necessary to confirm the nature of phage alterations and their association with intestinal diseases. The establishment of a comprehensive database and the use of valid model systems would pave the way for the identification of viral dark matter, complementing our knowledge of the mechanisms of bacteria–phage interactions (Camarillo-Guerrero et al., 2021). Understanding the intricate and precise regulatory roles of phages in maintaining gut health and homeostasis, whether through direct mechanisms or host-mediated pathways, will advance the accuracy of phage therapeutics applications.

## 5 Phage-related therapies

### 5.1 Phages versus antibiotics

PT employs bacteriophages isolated from common environmental reservoirs to specifically target and eradicate pathogens in human hosts, a concept regarded as dynamic drug therapy (Rossi et al., 2016; Divya Ganeshan and Hosseinidoust, 2019). Accumulating data suggest that PT can serve as a viable alternative or aid to antibiotic treatment (Keen and Adhya, 2015; El Haddad et al., 2019). The advantages of PT over conventional antibiotics are manifold:

1. Most phages could only infect specific genera of bacteria. Such a feature guarantees the elimination of the target pathogen, without overly affecting the commensal microflora.
2. Phages possess the capability to disrupt biofilm structures, which are notoriously resilient to antibiotic penetration (Gabisoniya et al., 2016a; Gabisoniya et al., 2016b).
3. As a bioactive substance, phage virulence is continuously evolving to overcome resistance developed by bacteria.
4. phages can penetrate physiological barriers, such as the blood–brain barrier, reaching infection sites the antibiotic concentration at the infection site is insufficient.

Interestingly, combining specific phage preparations with antibiotics has been shown to have higher bacterial killing efficiency and a lower mutagenesis rate of the bacterial population in multiple preclinical studies (Gordillo Altamirano et al., 2022) (Kamal and Dennis, 2015). There is even evidence that phage, despite losing its ability to directly combat bacteria, nonetheless plays a role in inhibiting the growth of bacteria that have developed increased resistance to antibiotics (Schooley et al., 2017). This phenomenon, called phage–antibiotic synergy (PAS), offers a multifaceted approach to combatting multidrug-resistant bacteria through various mechanisms.

Bacteria rapidly evolve countermeasures to resist external selective pressures by antibiotics and phages alike through modifications or loss of bacterial structures such as the capsule, fimbriae, or pili. At the same time, this resistance, termed a “trade-off”, presumably alters the functional properties of receptors, such as attenuated virulence, defects in host colonization, or altered antibiotic susceptibility, with a great physiological cost for bacteria (Goldhill and Turner, 2014; Torres-BarcelÃ and Hochberg, 2016; Oechslin et al., 2017). Chan and colleagues recently identified that bacterial phage resistance conferred by mutations of efflux proteins helps bacteria re-sensitize to co-administered antibiotics due to inefficient efflux. In subsequent clinical experiments, phage OMKO1 in synergy with Cefeffective forces a clinically relevant trade-off in bacteria and successfully prevents spread of drug resistance (Chan et al., 2016; Chan et al., 2018). Similarly, research by Altamirano et al. has demonstrated that phage-resistant variants, via phenotypic trade-offs, exhibit heightened susceptibility to antibiotic-induced killing, a mechanism that appears to be repeatable and predictable (Gordillo Altamirano et al., 2022). Besides, the presence of Polysaccharide depolymerizes in lysogenic phages enables the disruption of bacterial biofilms, facilitating antibiotic penetration and enhancing the bactericidal action (Pires et al., 2016b).

At the same time, the presence of antibiotics improved bacterial killing by phages. Previous data suggest that PAS is always accompanied by delayed lysis and bacterial filamentation, leading to increased phage production (Kim et al., 2018). In a study examining steps of the lytic cycle of phage HK620 in antibiotic-treated *E. coli* cultures, the researchers further noted that sublethal doses of antibiotics inhibited bacterial division, resulting in highly filamentous cells. Phages replicated rapidly within these elongated cells, which possessed larger surface areas, facilitating easier cell lysis (Bulssico et al., 2023).

It is noted that phage–antibiotic combinations may not always yield beneficial effects and can even result in antagonistic interactions. For instance, the effect of the ability of bacteriophages to eradicate biofilm was observed to decrease for combined exposure to phage T3 and kanamycin (Zuo et al., 2021). Theoretically, antibiotics rapidly kill pathogenic bacteria, phage replication rates are too low thus limiting their bactericidal effects (Petrovic Fabijan et al., 2020). Despite the potential benefits, the clinical translation of phage-antibiotic combinations poses challenges, as each combination’s effectiveness may be unique, and numerous unknown variables require further exploration. Determining the optimal order of bacteriophage–antibiotic treatment and the appropriate therapeutic dosage of antibiotics are areas that warrant additional investigation.

### 5.2 PT in the food industry

Foodborne disease poses a significant threat to public health, stemming from contamination by pathogens (bacteria, viruses, or parasites) or toxic chemical substances, and accounts for approximately 600 million illnesses worldwide annually (WHO, 2020). Bacterial pathogens mainly include *Salmonella* spp., *Campylobacter*, *E. coli* (mainly serotype 015:H7), *Listeria monocytogenes*, *Campilobacter jejuni*, and *Staphylococcus aureus*. These bacteria not only contribute to food spoilage but also pose infection risks in food-producing animals, resulting in economic losses and health hazards.

Traditionally, most bacterial killing is predominantly based on physical and chemical methods, such as heat pasteurization, HPP, or chemical sanitizers, with potentially cytotoxic effects and environmental contamination; others involve biopreservation techniques, primarily lactic acid bacteria. Phage biocontrol might be a promising new tool to eradicate foodborne bacterial pathogens due to its environmentally friendly, green, and safe. Sensory evaluation demonstrated that the color, taste, and appearance of food did not have significant differences after and before treatment with phage (Perera et al., 2015). Several phage development companies are presently marketing phage-based formulations as biocontrol agents for foodborne illnesses. These products, including ListShield™ and LISTEX™ P100 for *Listeria monocytogenes*, EcoShield™ for *E. coli* O157:H7, and Phageguard S for *Salmonella*, have been proven effective in eliminating contaminants from food and poultry products (Silk et al., 2012; Sillankorva et al., 2012; GutiÃ©rrez et al., 2017; MiguÃ©is et al., 2017). The utilization of phage formulations on live animals as a substitute for antibiotics resulted in significant efficacy against pathogenic bacterial infections, with concurrent observations of good tolerability and safety. In experiments with colostrum-fed calves, oral PT successfully prevented experimental diarrhea with enteropathogenic *E. coli* strains (Smith and Huggins, 1983). Subsequent investigations revealed that although phage-resistant bacteria emerged in the intestines of the calves, these strains, lacking the K antigen, did not induce diarrhea when reintroduced into healthy calves (Taylor and Roberts, 2005). Phages targeting *Clostridium perfringens* have demonstrated success in treating broilers with necrotic enteritis and exhibit stability across a range of temperatures and pH levels (Bae et al., 2021). Moreover, phages have potential utility in detecting bacteria in food and in the bacteriological control of food equipment (Jaglan et al., 2022). However, several plant and fish pathogens have demonstrated phage resistance (Sieiro et al., 2020). The limited knowledge of phage biology and infection dynamics in both solid and liquid food matrices also impedes the broader adoption of this technology.

### 5.3 PT in intestinal infectious diseases

Phage products were already being used in the former Soviet Union to prevent and treat enteric infectious diseases and wound infections during and after World War II. In 1963 and 1964, a large, randomized, double-blind, placebo-controlled clinical trial (n=30769) was performed to assess the safety and efficacy of oral administration of phages that target prophylaxis of infectious diseases including bacterial dysentery. In a 109-day follow-up of the children, dysentery in the phage-treated group was 3.8-fold lower than the placebo group through clinical diagnosis, and 2.6-fold lower as confirmed through culture (Sulakvelidze et al., 2001). A subsequent clinical study conducted in 1984 reaffirmed the efficacy of PT in reducing dysentery incidence (Anpilov and Prokudin, 1984). Since the 21st century, despite the advances in antibiotic treatments, dysentery outbreaks have persisted in regions with poor socio-economic conditions due to the emergence of antibiotic-resistant strains. The program about PT to treat dysentery was gradually restarted. For the *in vitro* experiments, the bactericidal rate of different phage cocktails against Shigella spp. can reach 90%, even over 99% (Mai et al., 2015; Shahin et al., 2021). Additionally, in animal models, comparable bactericidal effects to those of ampicillin were observed, with no disruption to the balance of the intestinal microbiota (Mai et al., 2015).

Since previous studies reported that almost all *C. difficile* phages are non-lytic, the reduced treatment effect of single *C. difficile* phages observed in *in vitro* assays poses a challenge to the application of PT for CDI (Li T. et al., 2020; Venhorst et al., 2022). To solve this problem, Nale et al. developed an optimized phage cocktail that completely cleared *C. difficile* after 24 h of culture in a batch fermentation model and was found to exert favorable effects on common major bacterial groups (Nale et al., 2018). In addition, new therapeutic perspectives including phage-derived proteins and engineering approaches are being explored to overcome this hurdle (Venhorst et al., 2022). For example, the temperate phage is genetically modified to become permanently lytic (Selle et al., 2020). Wang et al. expressed a recombinant prophage lysin (PlyCD) with vigorous lytic activity to colonize *C. difficile* in a mouse model of intestinal infection. The collaboration between vancomycin and PlyCD resulted in a significantly higher killing efficacy against *C. difficile (* Wang et al., 2015). Thus, therapeutic phages and their lysins could represent new weapons for combating CDI, either as standalone treatments or in combination with other antimicrobials, to eradicate the pathogen and prevent recurrence (Schmelcher et al., 2015). Phage resistance is another key concern. Phage-resistant strains of *C. difficile* have been observed in both *in vivo* experiments during PT and *in vitro* laboratory studies (Ramesh et al., 1999; Kirk et al., 2017).. Studies on resistant strain indicate that mutations in SlpA are a primary cause of bacterial resistance. This resistance comes with metabolic costs, such as the loss of virulence factors and increased sensitivity to antimicrobial peptides, which facilitates the effectiveness of antibiotics (Kirk et al., 2017).

Cholera is an infectious disease characterized by severe diarrhea, caused by the presence of toxic strains of V. *cholerae* bacteria. At present, the primary means for controlling cholera is vaccination due to its short incubation period and high mortality. Yen et al. demonstrated in mouse and rabbit models that oral administration of phages effectively killed pathogenic bacteria, reduced V. *cholerae* colonization, and prevented cholera-like diarrhea (Yen et al., 2017). In another experimental study, by collaborating with high salt which can increase the transcription level of the tolC gene, the expression of tolC as a phage receptor is increased, and thus VP3 Phage enhances the efficacy of eliminating biofilm-associated V. *cholerae* (Li et al., 2023). Phage prophylaxis opens a new promising path in controlling *Vibrio cholerae* infections, despite the bacterium’s evolved resistance mechanisms, including the use of outer membrane vesicles (OMVs) to neutralize lysogenic phages (Reyes-Robles et al., 2018).

To date, PT has demonstrated favorable outcomes in treating wound-associated infections and pulmonary infections, particularly as a substitute therapy for pan-drug-resistant bacterial infections. However, there is a notable scarcity of exploitable clinical data on gut infections, largely due to the intricate nature of the gut microbial community and limited understanding of infectious disease processes. Sarker et al. initiated a study on PT targeting *E. coli* in children with diarrhea in Bangladesh. Although the treatment effect did not reach statistical significance, their findings confirmed the overall safety of oral phage administration (Sarker et al., 2016). To better exploit the role of phage agents in preventing and treating outbreaks of enteric infectious diseases, it is necessary for some national public health agencies or the World Health Organization to take the lead in organizing the screening of infectious diseases that satisfy the favorable conditions for PT and to conduct the relevant clinical trials to prove their efficacy and safety.

### 5.4 PT suppresses inflammation through precision editing of the gut microbiota

Strains of adherent-invasive *E. coli* (AIEC) are opportunistic pathogens that can abnormally colonize the ileal mucous membrane and bind to the host adhesion receptor known as carcinoembryonic antigen-related cell adhesion molecule 6, thus increasing the inflammatory response (Bretin et al., 2018). Galtier et al. found that composite phage treatment exponentially decreased the number of AIEC bacteria and reduced colitis symptoms in conventional mice (Galtier et al., 2017). These findings suggest that specific phages can be utilized to kill targeted bacteria and reduce the incidence of invasive bacteria-exacerbated CRC. The similar conclusion was made from a recent study that evaluated the safety and efficacy of AIEC-targeted phage mixtures (EcoActive™). Researchers further determined that Long-term phage administration did not result in significant differences in the composition of bacterial communities of healthy mice, implying that its use is safe (TitÃ©cat et al., 2022). In another study with two enteropathogens (*E. faecalis* and *E. coli*) implicated in the development of IBD, Buttimer et al. observed a significant reduction in the number of bacterial cells, with a decrease of 1.1 log_10_ genome copies/mL for E. *faecalis* and 1.5 log_10_ genome copies/mL for E. *coli*, following phage treatment in an *in vitro* model. However, the potential of phage cocktails to reduce host numbers of proinflammatory bacteria was not demonstrated in a mouse model and phage use affected the relative abundance of other members of the intestinal microbiota (Buttimer et al., 2022).

*Fusobacterium nucleatum* (Fn) was shown to impede the host anticancer immune response by enlarging myeloid-derived immunosuppressive cells (MDSCs) and further dampening T-cell responses (Kostic et al., 2013). Reduction in MDSC amplification and precise clearance of Fn at tumor sites was achieved by combining Fn-specific binding of M13 phage with antimicrobial agents (Dong et al., 2020). A more recent study by Galtier et al. identified a clade of *Klebsiella pneumoniae* (Kp) strains implicated in the severity of IBD. Mice infected with clinical IBD-associated Kp strains showed increased intestinal inflammation. When *Klebsiella*-targeting phages were administered to Kp-2H7-colonized specific-pathogen-free mice, the Kp-2H7 bacterial burden was significantly reduced and led to an attenuation of inflammation. Such combinatorial phage treatments have been proven to have acceptable safety and stability profiles (Federici et al., 2022).

Duan et al. identified that cytolysin, a two-subunit exotoxin produced by *Enterococcus faecalis* (*E. faecalis*), exhibits eukaryotic cytolytic toxicity, contributing to liver damage (Cox et al., 2005; Duan et al., 2019). In patients with alcoholic liver disease, the proportion of *E. faecalis* (5.59%) was significantly higher than in healthy controls (0.023%). Notably, cytolytic *E. faecalis* strains exhibited a significant correlation with the severity and mortality of alcoholic hepatitis. Consequently, the researchers hypothesized that targeting cytolysin-positive *E. faecalis* with phages could reduce the risk of liver failure in alcoholic liver disease. To test this hypothesis, the researchers subjected mice either with *E. faecalis* overgrowth or wild-type controls to a chronic-binge ethanol diet. Subsequently, the former group received a phage cocktail targeting cytolytic *E. faecalis* via gavage, while the latter group received innocuous *Bacillus crescentus* phages. Compared to the control group, mice administered *E. faecalis* phage exhibited reduced inflammatory damage and steatosis in their livers. This improvement was accompanied by a decrease in the number of *E. faecalis* in the intestinal tract and a reduction in the level of cytolytic cytokines. Additionally, the researchers proposed lysostaphin as a potential prognostic biomarker for alcoholic hepatitis (Duan et al., 2019).

### 5.5 CRISPR antibacterials

Clustered regularly interspaced short palindromic repeats and associated proteins (CRISPR-Cas) systems is bacterial adaptive immune systems against DNA invasion. In the CRISPR system, functional spacers derived from viral DNA sequences and protospacer adjacent motifs (PAM) on either side for binding and targeting are included. As viral DNA enters its host cells, short segments of this exogenous DNA are inserted into the CRISPR locus of the bacterial genome. In the CRISPR system, functional spacers derived from invader sequences and protospacer adjacent motifs (PAM) on either side for binding and targeting are included. After translation and processing of these regions, mature CRISPR short RNAs (crRNA) are produced. After translation and processing of these regions, mature CRISPR short RNAs (crRNAs) are produced and then form a complex with Cas effector protein. When the same foreign nucleic acid reinfects, CRISPR RNA-guided Cas proteins degrade the viral DNA (De Sordi et al., 2019). The CRISPR-Cas9 system, known for its simplicity, is broadly utilized for precise targeted gene editing. Besides, the active Type I-C CRISPR-Cas system can specifically target phage DNA that possesses single and even multiple mutations (Soto-Perez et al., 2019). However, phages can defeat microbial defense mechanisms in multiple ways, including phage gene point mutations or deletions in protospacer and PAM sequences, phage DNA glucosylation, and encoding direct or indirect protein inhibitors of CRISPR-Cas (anti-CRISPR proteins) (Tao et al., 2018; Vlot et al., 2018). Tao and his team further demonstrated that phages with cytosine hydroxymethylation and glycosylation modifications in the CRISPR-edited regions can inhibit CRISPR-Cas9 cleavage, compared to unmodified phages. These CRISPR escape mutations accumulate at an astonishing rate. By the third-generation of plaques, nearly all plaques were composed of the mutant phage, compared to just 5% to 10% in the first-generation (Tao et al., 2017). Therefore, the CRISPR-Cas system aids bacteria in defending themselves against phages while simultaneously establishing a selective advantage for phages, highlighting the coevolutionary arms race between bacteria and bacteriophages.

Given the ability of temperate phages to self-replicate and transfer their own genes to host bacteria, researchers have proposed using CRISPR-Cas system-based genome editing tools as antibacterial agents (Kim et al., 2019; Yeh et al., 2022). The CRISPR-Cas systems targeting antibiotic-resistance genes are packaged into phage shells (phage membranes) or incorporated into the phage genome. Upon infecting the target host, these phages use the CRISPR-Cas system’s precise cutting capability to eliminate AMR genes, making the target bacteria susceptible to antibiotics once again. This approach uses phages as carriers and leverages defense mechanisms of host bacteria to create weapons against antimicrobial resistance (AMR). Bikard et al. demonstrated that delivery of CRISPR-Cas9 constructs via transduction of phagemids could be used in a sequence-specific manner to kill bacterial pathogens carrying AMR genes and remove plasmid-borne resistance genes in the mouse model of *S. aureus* skin colonization (Bikard et al., 2014). Control groups that were established to compare the efficacy of this new antimicrobial approach to traditional antibiotics revealed that the killing efficacy of CRISPR/Cas9-mediated targeting of *S. aureus* was better than that of other treatments, but worse than chloramphenicol. In another study, researchers engineered self-targeting capabilities into an endogenous type I-B CRISPR-Cas system in *C. difficile*. These phages were modified to exhibit reduced lysogeny and deliver lethal genome-targeting CRISPR arrays (Selle et al., 2020). Using this antimicrobial approach, *C. difficile* numbers decreased exponentially *in vitro*, and 90% of the self-targeted bacteria in colonies cultured from mice were killed.

Despite these encouraging results, several obstacles must be overcome when applying this technology. In addition to the risks associated with phage use, this technology is also limited by the potential risk of heterologous gene insertion. Ensuring Cas protein activity and minimizing bacterial evasion of CRISPR-Cas interference are also essential. Currently, most CRISPR-based antibacterials target single antibiotic resistance genes. Expanding the phage targeting range to include multiple plasmid or chromosomal sequences may help mitigate the emergence of resistant mutations (Bikard et al., 2014). Additionally, when targeting pathogens in the gut, this technology can be used to restore the sensitivity of native microbiota to pathogens, thereby indirectly killing the pathogens and minimizing disruption to the gut environment. We are confident that these engineered phage-derived antibacterials can be effectively used in clinical pathogen-phage systems, as most pathogens possess compatible temperate phages and CRISPR-Cas systems, which have been demonstrated to be robust and precise genome editing technologies.

## 6 Potential limitations of PT

### 6.1 Phage characterization

Most phages are limited to targeting a number of strains within bacterial species, posing challenges in eradicating clinical infections caused by multiple pathogenic bacteria using a single phage. A viable approach against this issue is using phage cocktails comprising a diversity of bacteriophages in phage preparations to infect a wider range of hosts. It is also possible to extend the host range of a single phage by genetic engineering techniques, such as genetic modification of the RNA binding proteins (RBPs) or phage tail-fiber proteins (Pires et al., 2016a). However, because the second method is often impractical and expensive in real-time settings, phage cocktails were more widely used to cover target bacteria. In addition, as temperate phages have both lytic and lysogenic cycles, providing virulence genes in bacterial pathogens, strictly lytic phages hold more promise in PT context. Notably, some virulent phage possesses an atypical infection strategy, which means cell lysis was not observed even using a high titer of phage lysate (Buttimer et al., 2017). Depth analysis of the phage genomes is, therefore, necessary to make sure its effectiveness in lysing bacterial cells as well as the absence of antibiotic resistance genes and virulence factors.

The rapid physical disruption of Gram-negative bacterial cells by phages can release a large number of endotoxins, which are among one of the most effective inducers of increased inflammation. *In vitro* experiments have shown that the concentration of endotoxins released following phage lysis of pathogenic bacteria is lower compared to that released by β-lactam antibiotics. This may be attributed to the phages’ faster cell lysis rate and their ability to reduce the occurrence of abnormal cells (Dufour et al., 2017). Nonetheless, it is crucial to recognize that endotoxin-mediated Gram-negative sepsis can have severe consequences, potentially leading to patient death, especially in cases of high inoculum infections and/or severe sepsis or septic shock. To address this issue, researchers have proposed using lysis-defective phages or non-lytic filamentous phages during phage selection, as well as eliminating phage lysis genes, to kill bacteria while minimizing bacterial lysis (Hagens and BLaosi, 2003; Hagens et al., 2004; Matsuda et al., 2005).

### 6.2 Phage immunogenicity

To effectively treat chronic infections, phages must endure *in vivo* for prolonged periods, evading eradication by the host immune system – a critical criterion for their therapeutic success. Based on the limited reports concerning the immune response to PT, factors such as phage dose, route of administration, frequency of administrations, level of phage solution purification, and treatment duration are significant determinants of the immunogenicity of human therapeutic phage preparations (Åusiak-Szelachowska et al., 2014; Szermer-Olearnik and Boratyaski, 2015; KaÅ°mierczak et al., 2021; Nick et al., 2022). Notably, among the various routes of administration, the oral route is associated with the weakest humoral immunity (Åusiak-Szelachowska et al., 2014). The early IgG response in the individual only appears to initiate mild neutralization of phage, and until relatively late in treatment, body mounts neutralizing antibodies against phage (Nick et al., 2022). Yet another study demonstrated that, regardless of the neutralizing properties of the anti-phage antibody against the administered phage, it did not influence the final therapeutic outcome (Åaczek et al., 2016). Thus, there is no evident correlation between the intensity of anti-phage humoral responses and the therapeutic outcome of PT (Åusiak-Szelachowska et al., 2021).

It is important to highlight that numerous patients receiving PT treatment exhibit diverse types of immune compromise, mainly due to organ transplantation and long-term antibiotic therapy. An important inquiry to explore is whether the immune response mounted against phages in PT contributes favorably to treatment outcomes. Should the immune response impede the effectiveness of PT, it prompts investigations into its impact on standardized PT in individuals with diverse degrees of immunodeficiency. Leung and Weitz and Roach et al. are optimistic about the immune system functioning. The former found that the introduction of phages can disrupt the immune evasion tactics employed by bacterial hosts, rendering them susceptible to the innate immune response (Leung and Weitz, 2017). This process known as “immunophage synergy” enhances the organism’s ability to recognize, attack, and eradicate pathogenic bacteria more effectively. The latter suggests that the combined effect of phage and host immune system can also be achieved through neutrophils recognizing phage-resistant variants (Roach et al., 2017). Further comparative analysis of phage treatment efficacy for acute infections in mice with varying immunodeficiencies and in immunocompetent mice showed that phage preparations effectively eliminated infection in immunocompetent mice but were unable to prevent the growth of phage-resistant mutants in MyD88-deficient mice and demonstrated complete ineffectiveness in lymphocyte-deficient mice (Roach et al., 2017). Interestingly, the equivalent conclusion was not replicated in clinical applications of PT. One case study used IV mycobacteriophage cocktail to successfully treat an immunosuppressed patient and no serum phage neutralizing activity was observed within 9 months of therapy onset (Dedrick et al., 2019). However, the same phage preparation in an immunocompetent patient has shown that strong neutralizing antibody responses in two months of treatment lead to failure of PT in a refractory *Mycobacterium abscessus* lung disease (Dedrick et al., 2021). It has been suggested that enhanced therapy efficacy may result from the utilization of a wider variety of therapeutic phages in immunodeficient hosts (Li G. et al., 2020).

### 6.3 Bacterial resistance

During the ongoing arms race between phages and their hosts, bacteria deploy multiple defense strategies at various stages of the phage life cycle to thwart phage invasion, including preventing phage adsorption and genome entry, blocking DNA transcription and replication (using tools including restriction-modification, CRISPR-Cas, and abortive infection systems), interphage immunity, and biomembrane protection (De Sordi et al., 2019). Meanwhile, new bacterial immunity mechanisms are continually being identified, such as cyclic GMP-AMP-mediated signaling and the Toll/interleukin-1 receptor (TIR) domain which has enzymatic NAD+ hydrolase activity (Doron et al., 2018; Cohen et al., 2019). The mechanisms of programmed genetic variation that counteract bacteriophages are typically induced through horizontal gene transfer, point mutations, and phase variation (PV). Compared to random point mutations, PV, based on the reversible high-frequency switching of bacterial phenotypes, enables pathogens to generate diversity more rapidly (Bikard and Marraffini, 2012). Additionally, phase variation can create bacterial population regions with different phage concentrations, facilitating discrete co-evolution of bacteria under varying phage pressures (Cayrou et al., 2021). Consequently, the rapid evolution of bacteria limits the effectiveness of single-dose PT, potentially prolonging treatment duration or leading to treatment failure. Bacterial resistance should be considered in any PT development process.

Although phage cocktails have been extensively embraced as a solution to confront this challenge, it is important to acknowledge their inherent risk of unintentional horizontal gene transfer. Therefore, ideal phages should be tailored to the individual patient. Methods such as BLAST-based analysis, CRISPR-based approaches, and the local K-mer strategy (LKS) have been developed to screen potential host organisms for query phages and accurately predict the connection between certain phages and bacterial mutants (Villarroel et al., 2016; Zielezinski et al., 2021; Lood et al., 2022). Furthermore, dedicated phage libraries targeting specific pathogens have been developed to enhance the efficiency of therapeutic selection (Touchon et al., 2017). Newly isolated phages can be added timely to the formulation when the resistant bacteria appear. Another strategy for the safe application of PT involves combining it with antibiotics (Oechslin et al., 2017). The application of viral gene products, such as endolysins, rather than intact phages, mitigates the horizontal transfer of bacterial virulence genes and curtails the emergence of phage-resistant bacteria (Schmelcher et al., 2012).

Phages can be improved via counter-resistance defense when confronted with the evolution of bacterial resistance mechanisms. Research has suggested that virulent phages accumulate point mutations that facilitate host jumping (adaptation to infect insensitive strains) in the gut of conventional mice, but not in dixenic mice or *in vitro* (Poirier and Vignuzzi, 2017). This implies that arms races between bacteria and phages lead to antagonistic co-evolution through a sustained buildup of genomic alterations. Some evidence suggests that RNAs encoded by bacteria and phages may influence one another’s post-transcriptional gene expression and control the lysis-lysogeny decision (Altuvia et al., 2018; Bloch et al., 2021). Phage mutant strains exhibit the capacity to infect diverse strains of the host bacteria, consequently extending the bactericidal range (Flores et al., 2011). Leveraging the ongoing struggle between phages and bacteria may offer insights to improve our efforts in combating microbial resistance.

### 6.4 The preparation of phage

Ensuring pharmaceutical-quality phages is essential before their clinical application, requiring several steps. Firstly, maintaining consistency and standardization of quality is paramount to guarantee the safety and efficacy of phages. Secondly, the therapeutic evaluation of phages must be determined, necessitating purity and stability in their preparation to eliminate confounding factors and obtain reliable data. The traditional method for phage purification involves a combination of polyethylene glycol precipitation, chloroform extractions, and cesium chloride (CsCl) gradient ultracentrifugation. While effective in removing endotoxin compared to alternative methods, this approach is unsuitable for medical use due to the use of harmful substances such as CsCl and chloroform. Besides, established phage production methods suffer from incomplete bacterial contaminants removal (Stacey et al., 2022). Hietala et al. compared the differences among purification methods in these contaminants removal in the production of phage preparations. They identified EndoTrap HD as the most suitable for clinical applications due to its ease of use, cost efficiency, and ability to produce highly purified phages (Hietala et al., 2019).

Therapeutic phage monitoring, which uses clinical response data to guide treatment, is suggested for integration into PT as a “best practice” standard. Furthermore, the compilation of clinical data, inclusive of phage pharmacokinetics, patient immune responses, the emergence of host resistance, and ecological monitoring of phages, will be aggregated to construct predictive models and evaluate the optimal administration, dosing and duration of phage treatment. This initiative seeks to bridge existing gaps in clinical data regarding the implementation of PT, while iteratively adjusting relevant parameters of marketed phage formulations to minimize disease risks and maximize healthy benefits (Bosco et al., 2023).

### 6.5 Parameters of the phage preparations

Variability in the biological properties of phage Preparations makes it difficult to gain insights into the pharmacokinetics and pharmacodynamics of phage products compared with a single compound. *In vivo* experiments, the half-life of phages was 2.3 h in plasma and up to more than 9 h in organs following a single intravenous bolus injection or during continuous infusion, keeping a relatively stable (Oechslin et al., 2017). However, the substantial variance in phage clearance between rat models and human patients is attributable to differences in clearance mechanisms and the titer-to-body weight ratio (Schooley et al., 2017). In the few clinical trials conducted, the phage concentration at the site of infection is often lower than expected, resulting in a slow self-amplification and low-efficiency bactericidal (Sarker et al., 2016). Conversely, excessive phage loads may precipitate the emergence of bacterial resistance (Chen et al., 2018). Studies are needed to establish reasonable dosing and frequency of administration as achievable to reliably treat each patient. In many scenarios, achieving a phage density of 108 phages/mL in the immediate vicinity of target bacteria may constitute an effective minimum target for bacterial eradication (Abedon, 2018; Danis-Wlodarczyk et al., 2021). In practice, a standard administration dose of a high phage dose is currently favored because of the high rate of bacterial reproduction and instability of phages *in vivo (* Sarker et al., 2016).

PT can be administered through various routes, including oral administration, nebulization inhalation, intravenous injection, and topical administration, with the latter two currently being more frequently used in clinical practice (**Table 2**) (Leung et al., 2019; Khalid et al., 2020). Tan et al. conducted a study to evaluate the biodistribution of phages and the host immune responses subsequent to intravenous injection, utilizing various purified phages in an animal model. Their findings unveiled considerable variances in the reduction of phage titers across different phage preparations following a single administration, coupled with a noteworthy decline in phage activity *in vivo* within a span of 72 hours. Upon repeated administration, in contradistinction to antibiotics, sustaining high titers of plasma phage activity proved unattainable, likely attributable to augmented non-specific phage clearance mechanisms. Furthermore, the researchers observed heightened pro-inflammatory signaling, specific antibody production, and endotoxin release ensuing from phage-mediated bacterial lysis *in vivo*, all contributing to an expedited clearance of phages (Tan et al., 2024).

**Table 2:** Compassionate PT.

Infection sites		Therapeutic targets	Host bacteria	Phage treatment	Administration route	Main outcome	Adverse drug reaction	Reference
lung		a 26-year-old cystic fibrosis (CF) patient	*Pseudomonas aeruginosa*	AB-PA01	intravenous	no exacerbation of disease	no observed	(Law et al., 2019)
lung		a 15-year-old patient with cystic fibrosis following bilateral lung transplantation	*Mycobacterium abscessus*	three-phage cocktail (Muddy, BPs33Δ HTH-HRM10, and ZoeJΔ 45)	intravenous	objective clinical improvement	no observed	(Dedrick et al., 2019)
lung		a 26-year-old man with CF and chronic lung infection	*Mycobacterium abscessus*	two engineered mycobacteriophages	intravenous	objective clinical improvement	no observed	(Nick et al., 2022)
lung		An 81-year-old patient with bronchiectasis	*Mycobacterium abscessus*	three-phage cocktail	intravenous	phage treatment failed	no observed	(Dedrick et al., 2021)
lung		3 lung transplant recipients (LTR)	*Pseudomonas aeruginosa* (case1 and case2) and *Burkholderia dolosa* (case3)	Case1: AB‐PA01 and Navy Phage cocktails 1 and 2 Case2: AB‐PA01 Case3: Single lytic phage	case1: nebulized and Intravenous case2 and case3: Intravenous	objective clinical improvement in case1 and case2 but insignificant in case3	no observed	(Aslam et al., 2019)
lung		a 32-year-old female with CF and underwent a double lung transplant	*Burkholderia multivorans*	Phage Bch7	nebulized and Intravenous	phage treatment failed	extreme leukocytosis beginning on day five of PT and rapid decline on day seven	(Haidar et al., 2023)
lung		a77-year-old woman with extensive, necrotizing, pulmonary pseudomonal infection	P. aeruginosa	AB-PA01	intravenous	objective clinical improvement	no observed	(Maddocks et al., 2019)
liver		a one-year-old girl with critically ill and three times liver transplanted	*Enterococcus faecium*	Phages EFgrKN and EFgrNG	intravenous	objective clinical improvement	no observed	(Paul et al., 2021)
pancreatic		a 68-year-old diabetic patient with necrotizing pancreatitis	*Acinetobacter baumannii*	nine-phage cocktail	intravenous and percutaneous	objective clinical improvement	no observed	(Schooley et al., 2017)
systemic infection		a 77-year-old man with postoperative infection with cerebritis, subdural and epidural empyema	*Acinetobacter* *baumannii*	five-phage cocktail	intravenous	lack of response to PT	transient hypotension	(LaVergne et al., 2018)
localized infection		a 42-year-old man with a trauma-related left tibial infection	*Acinetobacter baumannii* and *Klebsiella pneumonia*e	phages ϕAbKT21phi3 and ϕKpKT21phi1	intravenous	objective clinical improvement	no observed	(Nir-Paz et al., 2019)
localized infection		6 patients with intransigent diabetic toe ulcers	*Staphylococcus aureus*	staphylococcal phage Sb-1	topical	objective clinical improvement	no observed	(Fish et al., 2016)
localized infection		10 patients with Infected diabetic foot ulcers	*Staphylococcus*	anti-staphylococcal phage (ISP)	topical	9 patients benefit from PT	no observed	(Young et al., 2023)
localized infection		four patients with severe musculoskeletal infections	*-*	three patients used BFC 1 and one patient used Pyo bacteriophage	topical	objective clinical improvement	one patient developed local redness and experienced pain	(Onsea et al., 2019)
localized infection		a 76-year-old male patient of an aortic Dacron graft with associated aorto-cutaneous fistula	*Pseudomonas aeruginosa*	phage OMKO1	topical	no recurrent infection	no observed	(Chan et al., 2018)

### 6.6 Standards and regulation of phage preparations

Currently, despite the theoretical effectiveness of phage agents in targeting bacterial hosts, including drug-resistant strains, their large-scale production, commercialization, and utilization as an antibiotic alternative are still hindered by regulatory frameworks. The dynamic nature of phage cocktails, tailored to adapt to bacteria-phage antagonistic coevolution, does not align with today’s regulatory frameworks, which are traditionally geared towards static drug manufacturing (Sybesma et al., 2018). Moreover, the environmental risks stemming from high concentrations of phages and the limited awareness and understanding of phages as novel drugs exacerbate the challenges associated with PT (Meaden and Koskella, 2013). At present, there are no phage products authorized for sale globally, and only a select few countries have approved their use for emergency purposes. About the regulation area, local health departments and regulatory bodies should reform their current pharmaceutical regulatory concepts and set up a PT framework to comply with Good Manufacturing Practice (GMP). Several PT projects have been pioneered in Europe, Australia, and the United States. To assist in the implementation and improvement of the projects, phage therapeutics centers have been established (Aslam et al., 2020). In addition, several studies are looking for standardized treatment and monitoring protocols for PT, and there is a growing trend toward standardization of PT (Khatami et al., 2022). We look forward to seeing that commercial phage preparations can be approved in advance by the regulatory authorities for production and effective clinical application as traditional drugs.

### 6.7 Limitation in the gut

When faced with enteric pathogens, oral administration is usually performed. The focal points demanding consideration are the clearance of the phage during its transit through the gastrointestinal tract and the ensuing immune response of the organism upon its arrival in the gut. When phages are exposed to the highly acidic and anaerobic environment of the stomach, they have severely compromised survival, with less than 1% surviving feeding via drinking water (Han et al., 2022). Various methods, including polymer and liposome microencapsulation, gastric acid inhibitors, and neutralizing agents, have been explored to enhance the efficacy of phages administered orally (Vandersteegen et al., 2011; Sarker et al., 2012; Miadzybrodzki et al., 2017). For *P. aeruginosa* enterica, researchers have developed a tablet containing a phage titer of 2 × 10^7^ pfu/mL, meeting the Physical Properties Evaluation standards of the British Pharmacopoeia. This tablet exhibits minimal impact on phage titer during manufacturing processes and can be scaled up for mass production, offering a viable solution for the industrial preparation of various oral phage formulations (Khanal et al., 2021).

When phages arrive in the gut, the anaerobic environment and complex biocomposition may cause foreign phages to clear quickly. Besides, phages are constantly interacting with the epithelial tissues of the gut, breaking through the intestinal barrier into circulatory system. The presence of mucins in the mucosal layer can change bacterial physiology by enhancing virulence, boosting motility, and facilitating biofilm development. Nevertheless, these alterations represent a trade-off for the bacteria, which, in the end, benefits the phages in eradicating the host bacteria (Almeida et al., 2019). The effectiveness of phages is similarly restricted by intestinal immunity. Research demonstrates that the phage protein gp12 induces a strong increase in IgA production in the gut. When the levels of these specific IgA rise, the number of active phages detected in feces gradually diminishes or even vanishes (Majewska et al., 2015). These mechanisms might lead to phage titers not meeting the ideal therapeutic requirements. Specific locations within the intestine, such as intestinal crypts, biofilm, and the mucosal region of the ileum, serve as protected areas where bacteria can avoid being attacked by phages (Maura et al., 2012; Simmons et al., 2018). Lourenço et al. evaluated the replication capacity of three different phages of strain Mt1B1 in different intestinal sites in Mt1B1-colonized oligo-mouse-microbiota (OMM)12 mice. They observed that the mucosal parts of the gut had significantly lower phage titers and phage: bacteria ratios compared with the luminal parts (Lourenaso et al., 2020). In addition, biofilms of phage-resistant bacteria form a barrier between phages and their sensitive hosts, preventing them from damaging the susceptible portion of the bacterial community (Simmons et al., 2020). The heterogeneous spatial phenomenon of bacterial refuges within the mucosal layer that protects phage-sensitive bacteria from infection may explain the harmonious coexistence of virulent phages and host bacteria. At the same time, this phenomenon prevents invasion of therapeutic phage. It is worth noting that accessory genes responsible for human pathogenesis, carried by gut bacterial phages, may be delivered and expressed once the oral phage reaches the intestine, thereby raising the likelihood of developing underlying disorders (Ma et al., 2018). To maximize the antimicrobial effects of phages in the gut, Javaudin et al. suggested timely quantify viable phage particles supporting phage amplification as well as monitoring change of phages in order to administer promptly(at least 4h after the bacterial challenge) (Javaudin et al., 2021). Higher phage titers and more frequent dosing may also be required to cope with the rapidly changing environment in the gut.

## 7 Conclusions

Through the latest progress in metagenomic research, we are gradually demystifying the “gut dark matter,” with the discovery and isolation of crAssphage being among the most remarkable achievements. Moreover, the co-evolutionary arms race between gut bacteria and phages, along with phages’ direct and indirect immune responses, highlights the important role of phages in gut biology. However, the absence of a universal marker gene, difficulties in batch production, and the lack of adequate research instruments have put phage research in an initial stage (Sausset et al., 2020). To thoroughly understand the physiological transformations of bacteriophages in the intestinal environment and to clarify the complex interaction network involving bacteriophages, the microbiome, and their human hosts, the advancement of cutting-edge technologies, such as RNA-based methods, is crucial.

Over recent years there has been a growing recognition of the significance of human gut phages in both maintaining health and contributing to disease. Substantial differences in gut phages among individuals in both healthy and diseased states suggest a potential role for the phage community in various gastrointestinal diseases, including foreign infections, colitis, and metabolic disorders. However, our understanding of phage biology and the relationship between phages and host disease is still in its early stages. Further studies to confirm the role of phages in the etiology of diseases and elucidate their mechanisms of action may aid in disease treatment by manipulating these microbial communities. Furthermore, the marked differences in specific phage populations between healthy and diseased states may serve as an adjunct in disease diagnosis.

Additionally, PT offers a powerful tool for precisely targeting and eliminating harmful bacteria, with applications in food processing, clinical infections, and cancer-associated bacteria, offering an alternative to the overuse of antibiotics. Regrettably, PT continues to encounter multiple limitations and problems, and its standardization and commercialization are still in their infancy. At present, PT is not widely accepted in clinical settings, with most PT applications being compassionate uses after the failure of antibiotic treatments. To enable broader application of this technology, it is crucial to thoroughly investigate issues related to the phage physiology, immune responses to phages, phage resistance, the safety and efficacy of phage products, and their standardized application. When clinical conditions permit, we recommend using personalized phage cocktails or a combination of phages and antibiotics to ensure maximum benefit and minimal harm to clinical patients. Additionally, we must pay close attention to the CRISPR antibacterials that combine phages with the CRISPR-Cas system, although this technology is still in its early stages. As we gather more information on potential avenues for phage-mediated manipulation of bacterial communities and minimize risks and limitations to the use of living microorganisms, the development of phage-based treatments will move forward in human clinical studies.
